# Photoconduction and Electroluminescence of Copper (II) Protoporphyrin and Chlorin Cu-C-e6

**DOI:** 10.3390/ijms24043178

**Published:** 2023-02-06

**Authors:** Dmitry A. Lypenko, Alexey E. Aleksandrov, Andrey Yu. Chernyadyev, Sergey I. Pozin, Aslan Yu. Tsivadze, Alexey R. Tameev

**Affiliations:** Frumkin Institute of Physical Chemistry and Electrochemistry, Russian Academy of Sciences, Leninsky prosp., 31, bld. 4, 119071 Moscow, Russia

**Keywords:** copper (II) porphyrins, photoluminescence, charge carrier mobility, photoconduction, electroluminescence, organic light-emitting diode

## Abstract

Cu (II) protoporphyrin Cu-PP-IX and chlorin Cu-C-e6 were found to have both thin solid film formation and charge carrier transport abilities. In the layers deposited by resistive thermal evaporation, the mobilities of holes and electrons are on the order of 10^−5^ cm^2^ V^−1^ s^−1^. Organic light-emitting diodes incorporating the dye molecules as emitting dopants demonstrate electroluminescence in the UV and near-IR ranges.

## 1. Introduction

Porphyrins are known as a class of cyclic aromatic hydrocarbons that possess a set of unique properties, such as the high light absorption in the UV spectral range [1], high charge carrier mobility [2], luminescent properties [3], and the photogeneration of singlet molecular oxygen [4]. Their physical and chemical properties depend to a high degree on the nature of the metal cation associated with the aromatic ring of the porphyrin molecule through metal–nitrogen coordination bonds [3].

This is especially evident in the luminescent properties of the porphyrin molecule. Depending on the nature of the metal cation associated with the porphyrin cycle, phosphorescence (Pd^2+^, Pt^2+^ cations) [5,6], fluorescence (Mg^2+^, Al^3+^ cations) [7,8], and fast and delayed fluorescence (Sn^4+^) dominate in luminescence [9]. The suppression of the luminescent properties of the porphyrin molecule can be achieved by binding the Ni^2+^ cation [10]. For this reason, Ni porphyrinates are an active component of composites in organic photovoltaic devices [3]. However, all the above-listed metal cations have a closed electron shell, i.e., being diamagnetic metal cations. Metal cations with an open electron shell, such as Cu^2+^, VO^2+^, Cr^3+^, when bound to the porphyrin cycle, add to it a number of special luminescent properties associated with the fact that the unpaired electron of the paramagnetic metal cation interacts with the triplet excited states of the porphyrin molecule, resulting in energy-unequal tripdublet and tripquartet excited electronic levels from which radiative phosphorescence processes are realized [11].

We have previously investigated the film-forming ability of Cu (II) tetraphenyl porphyrinate (CuTPP) and Cu (II) tetrafluorenylporphyrinate (CuTFP), the charge carrier mobility and luminescent properties of the films deposited by the resistive thermal evaporation (RTE) technique, and the electroluminescence of these porphyrins in the poly-N-vinylcarbazole (PVK) matrix. The results of the study showed a high charge mobility and luminescent properties in their RTE films, and also the electroluminescence in the near-IR range [2].

**Figure 1:**
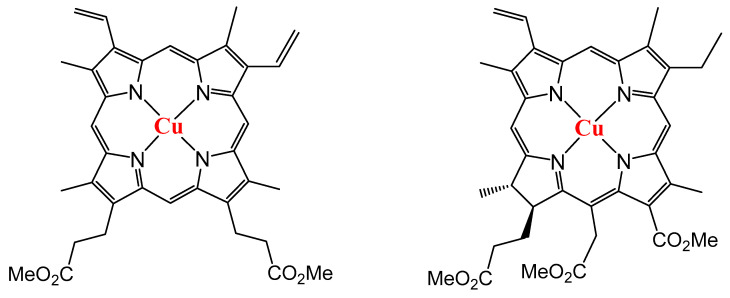
The structure of Cu (II) protoporphyrin Cu-PP-IX (**left**) and chlorin Cu-C-e6 (**right**).

In this work, we continue the research related to the formation of Cu (II) porphyrins films by the RTE method, the analysis of their charge carrier mobility, and the study of the photo- and electroluminescent properties. A relatively inexpensive semi-synthetic Cu (II) protoporphyrin (Cu-PP-IX) and Cu (II) complex with modified chlorin-e6 (Cu-C-e6) are selected as the objects of the research (Figure 1). These compounds differ greatly in structure from tetraphenylporphine derivatives; they have (a) no aromatic substituents in the meso-positions of the porphyrin ring and (b) a lower symmetry of the molecule compared to tetraphenylporphine derivatives. The latter is reflected in the electronic structure of these copper complexes and, accordingly, may lead to differences in the electrochemical and luminescent properties of the semisynthetic Cu (II) porphyrin and chlorin compared to tetraphenylporphine derivatives. Moreover, the molecules of the modified free base of chlorin-e6 (C-e6) and Cu-C-e6 have to manifest the effect of the optical circular dichroism and circular polarized luminescence as well due to two stereocenters (carbon atoms) are located inside the molecules of C-e6 and Cu-C-e6 (Figure 1). The organic light-emitting diodes (OLEDs) using these Cu (II) protoporphyrin and chlorin are fabricated and tested for the first time.

## 2. Results and Discussion

### 2.1. UV-Vis and IR Spectroscopy

**Figure 2:**
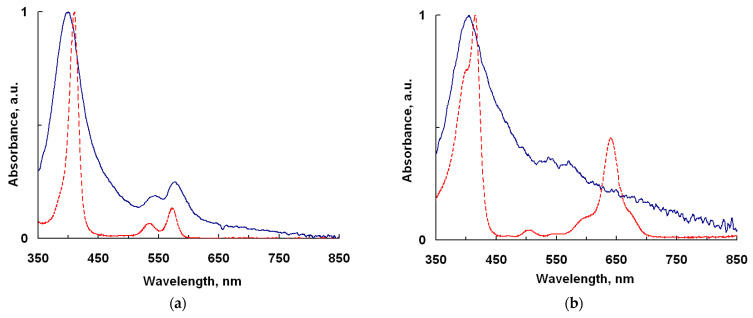
Cu-PP-IX (**a**) and Cu-C-e6 (**b**) normalized absorption spectra of the thin films (blue) deposited onto glass substrate by the RTE method and the solutions in chlorobenzene (red).

**Figure 3:**
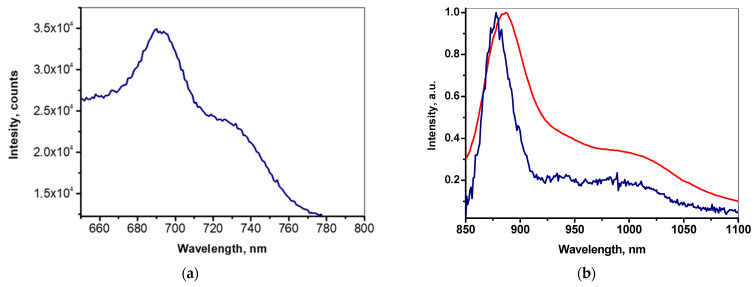
Photoluminescence spectra of the (**a**) Cu-PP-IX RTE film (excitation at 405 nm @ 295 K) and (**b**) Cu-C-e6 dispersed in PS film at 298 K (red line) and at 77 K (blue line), excitation at 400 nm.

In the UV−Vis absorption spectra of the Cu-PP-IX and Cu-C-e6 solid layers deposited by the RTE, a Soret band around 375 nm and a Q-band in the range of 500−600 nm are exhibited (Figure 2). These absorption bands are characteristic for Cu (II) porphyrins and indicate that the Cu-PP-IX and Cu-C-e6 molecules are not degraded in the RTE process. Under UV excitation, the corresponding photoluminescence spectra of the dyes demonstrate maxima in the near-IR range (Figure 3). The Cu-PP-IX film exhibits a weak luminescent (Figure 3a) similar to that observed earlier for CuTPP and CuTFP films [2]. However, the Cu-C-e6 film does not exhibit luminescence, while the Cu-C-e6 molecules dispersed in a polystyrene film exhibit intense photoluminescence in the near infrared at both 298 and 77 K (Figure 3b).

### 2.2. Stability of the Dye Molecules during Thermal Evaporation

The stability of the chemical structure of the Cu-PP-IX and Cu-C-e6 compounds after RTE on the substrate was controlled by washing the substance off the substrate with chlorobenzene. The UV–Vis absorption spectra of the resulting solutions were compared with those for the initial Cu(II) protoporphyrin and chlorin in a chlorobenzene solution. The absorption spectra turned out to be completely identical for both Cu-PP-IX and Cu-C-e6 (Figure 2).

Additional studies of the thermal stability of dye molecules were made by IR spectroscopy and X-ray spectroscopy using chlorin Cu-C-e6, since this dye was subjected to thermal sublimation at a temperature (390 °C) higher than that for the protoporphyrin Cu-PP-IX (360 °C). For Cu-C-e6 washed off from the substrate with chlorobenzene after RTE deposition, the IR spectrum was measured on a KRS-5 plate and compared with the spectrum of the initial Cu-C-e6, which was also deposited from a solution in chlorobenzene on a KRS-5 plate (Figure S2). The identity of the IR spectra in addition to the UV/vis spectra (Figure 2b) confirms the invariance of the chemical structure of chlorin Cu-C-e6 after deposition on the substrate by the RTE method. The IR spectra contain a set of lines in the region of 2750–3000 cm^−1^, which, apparently, refer to the CH_2_ and CH_3_ vibrations of the Cu-C-e6 molecule, the intense absorption lines at 1722 and 1736 cm^−1^ obviously refer to ester groups of the Cu-C-e6 molecule, and a complex set of absorption lines in the range of 600–1600 cm^−1^ probably corresponds to different types of vibrations of the chlorin cycle, which was previously observed for other metal chlorinates [12]. Yet, attempts to assign lines in the range of 600–1600 cm^−1^ for metal chlorinates [12] were not made as widely and as in detail as it was performed for the Raman spectra of metal chlorinates and porphyrinates [1]. The lines in the region 2250–2400 cm^−1^ correspond to the absorption of the CO_2_ molecules contained in the air. The stability of the chlorin molecules after the RTE processing is also confirmed by the XPS spectra, which are actually the same for the initial chlorin and after its thermal sublimation in Figures S3 and S4, respectively. In particular, the shapes of the Cu2p band substantially show that the spectra (Figure S5) reflect the same compound.

### 2.3. Dye Solid Layers

**Figure 4:**
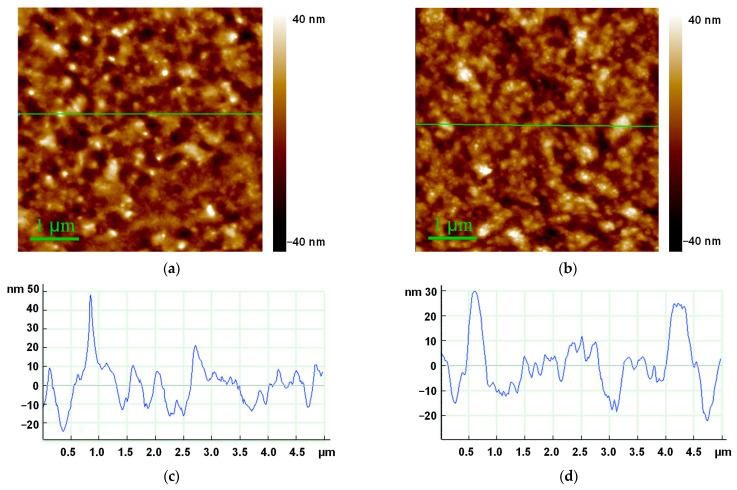
Atomic force microscopy (AFM) images of 65 nm thick films of (**a**) Cu-PP-IX and (**b**) Cu-C-e6; (**c**,**d**) height profiles along the indicated green lines shown in panel (**a**,**b**), correspondingly.

**Figure 5:**
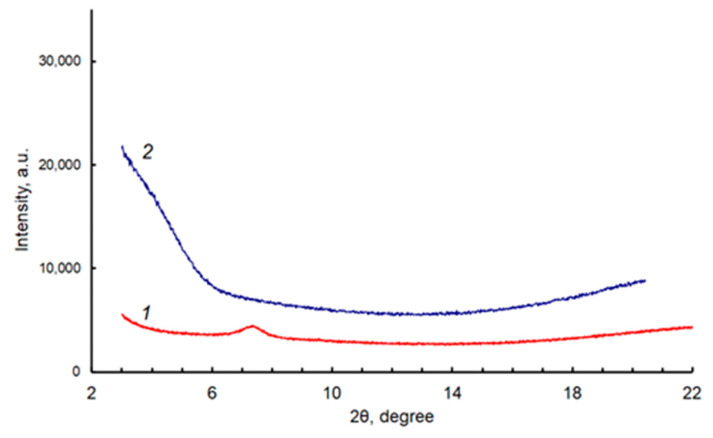
XRD patterns for the RTE-deposited layers of Cu-PP-IX (1, red line) and Cu-C-e6 (2, blue line).

The RTE-deposited layers of the compounds were characterized by AFM, SEM, and XRD methods. SEM and AFM images (Figure 4 and Figure S6) are in agreement with each other, but AFM provides a better contrast (resolution in z-direction) for the studied samples. The surfaces of the Cu-PP-IX and Cu-C-e6 layers are relatively smooth when the root-mean-square roughness (RMS) does not exceed 10 nm (Figure 4). Both have a similar granular morphology, but the grains are smaller and more clearly defined for the Cu-C-e6 layer. XRD spectra show that the RTE-deposited Cu-PP-IX and Cu-C-e6 layers are almost amorphous (Figure 5).

### 2.4. HOMO and LUMO Levels

**Figure 6:**
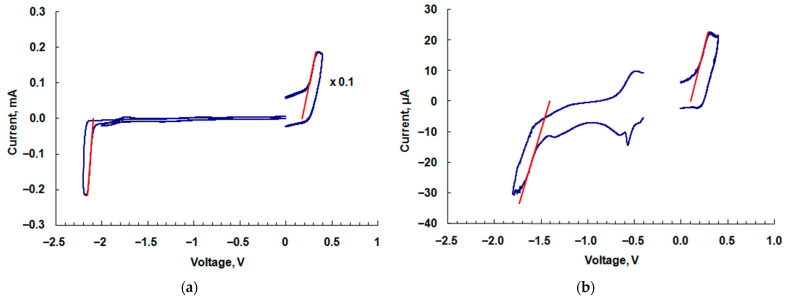
Cyclic voltammograms of (**a**) Cu-PP-IX and (**b**) Cu-C-e6 (blue curves). The red lines show the tangents to the curves. The accuracy of the CV experiments is ±0.02 V.

From the tangents (red lines) to the cyclic voltammogram (CVA) curves of Cu-PP-IX (Figure 6a) and Cu-C-e6 (Figure 6b), oxidation and reduction potentials are calculated and the corresponding data for the HOMO and LUMO levels are listed in Table 1. The data for PVK are available in reference [13].

### 2.5. Charge Carrier Mobility

Measured by the linear increasing voltage (CELIV) technique in the MIS-CELIV mode [14], the hole and electron mobilities in the RTE-deposited Cu-PP-IX и Cu-C-E6 layers are on the order of 10^−5^ cm^2^ V^−1^ s^−1^ (Table 2), which is higher than that in the CuTFP and CuTPP films by about one order of magnitude [2]. Evidently, in such spatially and energetically disordered media, the charge transfer occurs by hopping between the molecules and the LUMO and HOMO energy states serve as the transporting sites for electrons and holes, respectively [15]. Since the core of the considering Cu-PP-IX, Cu-C-E6, CuTFP, and CuTPP molecules are similar, the electron densities on the HOMO and LUMO levels are distributed over the core part of these molecules in the same way as in CuTFP and CuTPP [2]. Based on this, we consider that the short lateral moieties of the Cu-PP-IX and Cu-C-E6 molecules promote a closer arrangement of the molecules in the films than in the CuTFP and CuTPP films, and, as a consequence, improve the intermolecular transfer of the charge carriers.

PVK (Figure S1) is a conventional host material possessing mobile holes and a good film-forming ability when depositing from a solution. The hole mobility of 1.7 × 10^−5^ cm^2^ V^−1^ s^−1^ was measured in PVK; this value is 4 ÷ 5 times greater than that reported elsewhere [16]. A possible reason for the discrepancy is related to contacts in an MIS device [17]. Anyway, PVK is a suitable material to use as a host for the studied Cu porphyrin and Cu chlorin due to their compatible energy levels (Table 1).

### 2.6. Photoconduction

**Figure 7:**
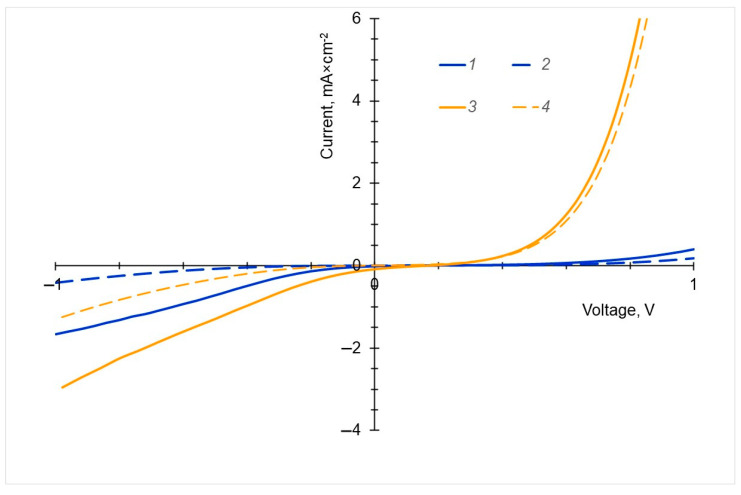
*I*–*V* curve of photocurrent (1, 3) and dark current (2, 4) for diodes based on the Cu-PP-IX (1, 2) and Cu-C-e6 (3, 4) dyes.

The photoconductivity of the new dyes was investigated on ITO/PEDOT:PSS(45 nm)/dye(40 nm))/C_60_(20 nm)/BCP(7.5 nm)/Al devices with the RTE-deposited dye layers. The *I*–*V* curves of the photocurrent and dark current are shown in Figure 6. At a reverse bias (quadrant III), the photocurrent in the device based on Cu-C-e6 (Figure 7, curve 3) is greater than in the device with Cu PP IX (Figure 7, curve 1). Since the electron and hole mobilities in the films of both dyes are comparable in magnitude (Table 2), the increased photocurrent in the device based on Cu-C-e6 is obviously due to the higher quantum yield of the photogeneration of the charge carriers in Cu-C-e6 than that in Cu PP IX. It is also important to note that with a forward bias (quadrant I), the dark current in the Cu-C-e6-based device (Figure 7, curve 4) is much higher than in the diode based on Cu PP IX (Figure 7, curve 2), i.e., the Cu-C-e6 can also be exploited in OLEDs. The reduced symmetry of the chlorin molecule compared to porphyrin leads to an increase in the dipole moment and the removal of the level degeneracy. As a consequence, this resulted in a better intrinsic charge generation and photogeneration followed by the larger photocurrent in chlorin-based photodiode.

### 2.7. Electroluminescence

**Figure 8:**
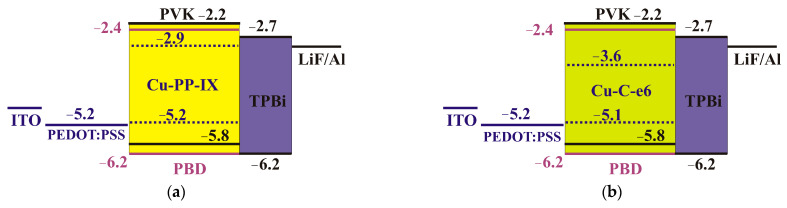
Energy diagram for the OLEDs involving (**a**) Cu PP IX and (**b**) Cu-C-e6. Energy levels for PVK [13], PEDOT:PSS [19], PBD [19], and TPBi [20] are from the literature.

Electroluminescent composites based on PVK:PBD with Cu(II) porphyrin and chlorin were prepared, with PVK serving as the film-forming matrix and PBD (Figure S1) serving as an electron transporting dopant since its electron mobility of 2.0 × 10^−5^ cm^2^ V^−1^ s^−1^ [18] correlates well with the hole mobilities in the PVK and Cu(II) porphyrins films. The HOMO level, the LUMO level, and the work functions of these materials are shown in the band diagram of the OLEDs (Figure 8). The HOMO−LUMO levels of the composite compounds (Table 2) match reasonably well with those of PEDOT:PSS, a typical hole-transporting layer, and TPBi, an electron-transporting (hole blocking) layer. In OLED, it is energetically favorable for the injected holes from PVK and for the electrons from PBD to transfer to the dye molecules with a subsequent radiative recombination.

**Figure 9:**
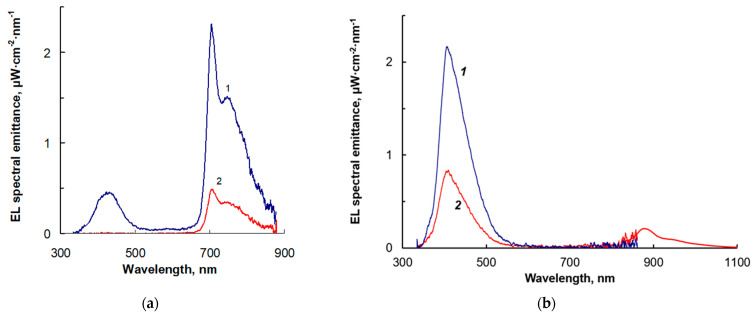
EL spectra of the OLEDs based on the PVK:PBD:dye composites with (**a**) Cu-PP-IX of the concentration of 1 wt.% (1) and 5 wt.% (2) at an applied voltage of 25 V, and (**b**) Cu-C-e6 of the concentration of 1 wt.% (1) and 2 wt.% (2) at an applied voltage of 15 V.

The electroluminescence (EL) spectra of the OLEDs with Cu-PP-IX exhibit maxima at 705 nm with a shoulder towards IR wavelengths and at 428 nm, while the EL intensity of the former is greater than that of the latter (Figure 9a). The OLEDs with Cu-C-e6 irradiate in the IR and UV ranges with EL maxima at 877 and at 405 nm, respectively, while, unlike the Cu-PP-IX-based OLEDs, the EL intensity of the former is less than that of the latter (Figure 9b), and it is PVK that emits in the UV (Figure S7).

We suggest that PVK, besides the hole transporting to the dye molecules, can promote the emission in the IR range when the dye molecules absorb the EL emission from PVK with subsequent photoluminescence in the near-IR range (Figure 3). However, the following processes are more likely: (1) excitons formed in the PVK:PBD composite transfer their energy to the Cu porphyrins, followed by the luminescence, (2) the Cu porphyrin molecules capture injected charge carriers, and after the approach of the opposite charge carriers, radiative recombination occurs. Despite the similar light absorption of the dyes in the emission band of PVK (400–500 nm), the processes involving more planar and aromatic Cu-PP-IX molecules are more efficient than those involving Cu-C-e6 molecules, as follows from the comparison of the EL intensities in the near-IR range with UV (Figure 9).

The EL emitting decreases with increasing the dye concentration (Figure 8). We believe this is due to the porphyrin molecules turning to be involved in the charge carrier’s transport. As the probability of the charge carrier transfer between neighbor molecules is known to increase with decreasing the average intermolecular distance *R*, the charge mobility increases as *µ*~exp(-*R/a*), where *a* is the wavefunction decay constant. At an increased mobility, part of the injected electrons and holes move through the EL active layer, avoiding the bimolecular recombination in the bulk and distribute in the vicinity of the anode and cathode, respectively. At a relatively high dye concentration in the composite, the average distance *R* between the dye molecules decreases and the probability of the charge carrier transfer between them increases, so the charge mobility increases. This leads to the disruption of the uniform distribution of the electrons and holes in the bulk of the EL layer: the concentration of the electrons and holes is higher at the anode and the cathode, respectively. Therefore, the recombination space narrows, and the radiation intensity decreases.

## 3. Materials and Methods

### 3.1. Synthesis of Compounds

The free base of protoporphyrin IX H2PP-IX was from Sigma-Aldrich (Burlington, MA, USA) and the free base of chlorin-e6 was from the Russian Technological University RTU MIREA. The structure and purity of the compounds were confirmed by NMR, UV/Vis spectroscopy and luminescent spectroscopy.

For the synthesis of Cu (II) protoporphyrin (Cu-PP-IX), 15 mg of the free base of protoporphyrin IX was dissolved in 25 mL of methylene chloride and a solution of 8 mg of Cu (II) acetate in 20 mL of ethanol was added to the resulting solution. The solutions were mixed, and the resulting solution was stirred at 40 °C for 30 min. The solvents were removed in a vacuum and the product was dissolved in methylene chloride and purified by column chromatography from traces of the original protoporphyrin IX. The yield of Cu-PP-IX was 16 mg (98%). The structure of the resulting Cu-PP-IX compound was confirmed by MALDI TOF mass spectrometry and UV/Vis spectroscopy in a chlorobenzene solution.

For the synthesis of Cu (II) chlorin-e6 (Cu-C-e6), 15 mg of the free base of chlorin-e6 was dissolved in 30 mL of methylene chloride and a solution of 7 mg of Cu (II) acetate in 20 mL of ethanol was added to the resulting solution. The solutions were mixed and the resulting solution was stirred at 35 °C for 40 min. The solvents were removed in a vacuum. The product was dissolved in methylene chloride and purified by column chromatography from traces of the original chorine e6, and the solvent was removed in vacuum. The yield of Cu-C-e6 was 16 mg (97%). The structure of the resulting Cu-C-e6 compound was confirmed by MALDI TOF mass spectrometry and UV/Vis spectroscopy in a chlorobenzene solution.

### 3.2. Photoluminescence Spectra

The photoluminescence spectra of the films were recorded on a modular spectrofluorometer Horiba Jobin Yvon S.A.S. (Kyoto, Japan) A xenon lamp with a power of 450 W was a source of the exciting light; an R928 photomultiplier and an InGaAs near-IR detector were used to detect the signals. The angle between the film surface and the excitation beam was 30°.

### 3.3. Cyclic Voltammetry

Cyclic voltammetry measurements and the calculation of the LUMO and HOMO energy levels were performed in the same way as described in our previous articles [2,21]. The obtained values of the HOMO and LUMO energy levels are listed in Table 1. Based on the data obtained, the functional layers of OLEDs and the devices for measuring the mobility of the charge carriers were prepared.

### 3.4. Resistive Thermal Evaporation (RTE)

The RTE procedure for depositing Cu(II) porphyrins thin films was described in detail earlier [2]. Cu (II) protoporphyrin and Cu (II) chlorin were evaporated at a crucible temperature of 360 and 390 °C, respectively. The RTE-deposited layers were impurity-free since the pressure in the vacuum chamber during their deposition remained unchanged.

### 3.5. Atomic Force Microscopy (AFM)

AFM observations were carried out in the tapping mode in air. Silicon cantilevers with spring constants of 5–40 N/m and resonant frequencies of 150–350 kHz were used.

### 3.6. Infrared (IR) Spectroscopy

The IR spectra were recorded using a Perkin Elmer Spectrum GX Fourier spectrometer (Waltham, MA, USA); the substances were deposited by a drop cast solution in chlorobenzene onto a KRS-5 plate.

### 3.7. X-ray Photoelectron Spectroscopy (XPS)

XPS were performed using a «PREVAC EA15» electron spectrometer (Rogów, Poland). AlKα (hν = 1486.6 eV, 150 W) was used as a primary radiation source. The pressure in the analytical chamber did not exceed 5 × 10^−9^ mbar. The binding energy (BE) scale was pre-calibrated using the positions of Ag3d5/2 (368.3 eV) and Au4f7/2 (84.0 eV) from silver and gold foils, respectively. The powdered catalyst samples were supported onto double-sided conducting scotch tape. To take into account the effect of surface charging, the C1s at Eb = 284.8 eV from the carbon contamination was used as an internal standard.

### 3.8. X-ray Powder Diffraction (XRD)

The X-ray diffraction patterns were acquired with Empyrean (PANalytical B.V., Eindhoven, The Netherlands) diffractometer equipped with a 1-D position-sensitive X’Celerator detector and Ni-filtered Cu Kα-radiation in Bragg–Brentano geometry.

### 3.9. Scanning Electron Microscopy (SEM)

The SEM images of the RTE-deposited dye layers were obtained using a Jeol JBM-7600F workstation (Tokyo, Japan) with a field emission cathode (high vacuum, 5 kV, SE).

### 3.10. Charge Mobility Measurements

Using the CELIV (carrier extraction by linearly increasing voltage) technique, the transient current in thin films was measured [14]. The CELIV current is caused by the movement of non-equilibrium charge carriers, which experiences a repeated capture and is released from traps during the drift from one electrode to the counter electrode. The details of the CELIV setup were described earlier [2,22]. In the work, we used the MIS-CELIV version, which makes it possible to record the current of the monopolar charge carriers. For the device’s preparation, a glass substrate with a 110 nm thick ITO layer (Kaivo Co.) was preliminarily cleaned and treated with UV radiation. A SiO_2_ layer 70 nm thick was deposited on top of the ITO by magnetron sputtering (the SiO_2_ layer excludes the injection of charge carriers of the opposite sign into the film under study). A dye layer with a thickness of 90 nm and an aluminum counter electrode were successively deposited through the mask by the RTE method. A PVK film with a thickness of 800 nm was deposited by drop casting a polymer solution in chloroform with a concentration of 10 mg/mL.

In the CELIV experiment, the time *t_max_* at which the conduction current reaches its maximum value was determined on the transient curve. The charge carrier mobility *μ* was calculated according to the equation:

$$\mu =\frac{2d^{2}}{3At_{max}^{2}(1+0.36\frac{\Delta J}{J(0)})}$$

where *d* is the film thickness, *A* is the voltage ramp, *J*(0) is the capacitance current, and Δ*J* is the conduction current at the time *t*_max_.

### 3.11. OLED Fabrication and Characterization

The fabrication of OLEDs and measuring their EL characteristics were described in detail in the previous article [2]. The hole transport layer of poly(3,4-ethylenedioxythiophene): poly(styrene sulfonate) (PEDOT:PSS, Clevios P VP AI4083; Heraeus, Hanau, Germany) was spin-coated onto the ITO/glass substrates and dried at 120 °C for 20 min. The substrates were then moved to an Ar-filled glovebox for the further fabrication processes.

The EL spectra of the OLEDs were measured with an AvaSpec 2048 fiber-optic spectrofluorometer and Fluorolog 3 instrument (Horiba Jobin Yvon S.A.S.; Kyoto, Japan) equipped by InGaAs detector for the near-IR range (800–1600 nm). In OLEDs with a composite containing 1 wt.% Cu-C-e6, the EL spectrum could not be recorded due to a very weak signal in the spectral range of 850–1100 nm. With an increase in the concentration of Cu-C-e6 in the composite up to 5%, the substance precipitated.

## 4. Conclusions

We have studied electroluminescence in OLEDs based on a composite containing a hole-transporting PVK and an electron-transporting PBD doped with 1–5 wt. % either Cu (II) protoporphyrin Cu-PP-IX or chlorin Cu-C-e6 for the first time. The devices exhibited UV and IR EL emission spectra, with the IR emission of the chlorin Cu-C-e6 containing OLEDs spanning up to 1100 nm. Although the chlorin shows increased I-V characteristics compared to the protoporphyrin in the photodiode’s structure, the protoporphyrin is more promising for the OLEDs development, for example, in a PVK matrix. The protoporphyrin manifests itself as an effective exciton energy acceptor transferred from PVK:PBD, because OLEDs exhibit enhanced emissions in the near-IR range than in the UV range of the spectrum. The EL intensity of the OLEDs depends on the concentration of the dye in the composites; therefore, further developments require the optimization of the concentration. The charge carrier mobility in the Cu-PP-IX and Cu-C-e6 solid layers has also been measured for the first time. Both materials possess reasonable electron and hole mobilities of the order of 10^−5^ cm^2^ V^−1^ s^−1^. These results show the prospects of using the studied dye molecules as promising materials for thin film optoelectronics. For example, the studied Cu (II) protoporphyrin and chlorin can be used in near-IR OLEDs instead of cyanine dyes with long methine chains, as reported in [23], which are known as low stable luminophores [24].

## Figures and Tables

**Table 1:** Energy of the HOMO and LUMO levels.

Molecule	HOMO, eV	LUMO, eV	Gap, eV
Cu-PP-IX	−5.213	−2.940	2.273
Cu-C-e6	−5.138	−3.630	1.508
PVK	−5.8	−2.2	3.6

**Table 2:** Charge carrier mobility (cm^2^ V^−1^ s^−1^) in Cu(II) porphyrins solid layers *.

Material	Holes	Electrons
Cu-PP-IX	(4.4 ± 0.4) × 10^−5^	(3.2 ± 0.3) × 10^−5^
Cu-C-e6	(2.0 ± 0.3) × 10^−5^	(4.8 ± 0.4) × 10^−5^

* Calculated from 10 replicates, the confidence level is 95%.

## Data Availability

Data are contained within the article and Supplementary Materials.

## Supplementary Materials

The supporting information can be downloaded at: https://www.mdpi.com/article/10.3390/ijms24043178/s1.
