# Supplementary material for: Photoconduction and Electroluminescence of Copper (II) Protoporphyrin and Chlorin Cu-C-e6
Source: Int J Mol Sci. 2023 Feb 6;24(4):3178. doi: 10.3390/ijms24043178 (PMC9964107; doi:10.3390/ijms24043178)
Supplement: Supplementary file 1 [file ijms-24-03178-s001.zip › ijms-2137379-supplementary.pdf]

Photoconduction and Electroluminescence of Copper (II) protoporphyrin and chlorin Cu-C-e6

Dmitry A. Lypenko, Alexey E. Aleksandrov, Andrey Yu. Chernyadyev, Sergey I. Pozin, Aslan Yu. Tsivadze and Alexey R. Tameev*

Frumkin Institute of Physical Chemistry and Electrochemistry, Russian Academy of Sciences, Leninsky prosp., 31, bld.4, Moscow 119071, Russia

**Corresponding author e-mail address: tameev@elchem.ac.ru (A. R. Tameev)*

Supporting Information

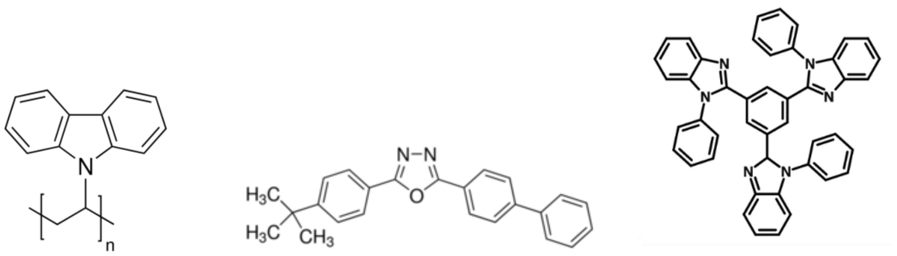

PVK PBD TPBi

Figure S1. Chemical structure of poly-N-vinylcarbazole (PVK) served as a matrix, 2-(4-tert-Butylphenyl)-5-(4-biphenylyl)-1,3,4-oxadiazole (PBD), and 2,2',2"-(1,3,5-Benzinetriyl)-tris(1-phenyl-1-H-benzimidazole) (TPBi) served as electron transporting components.

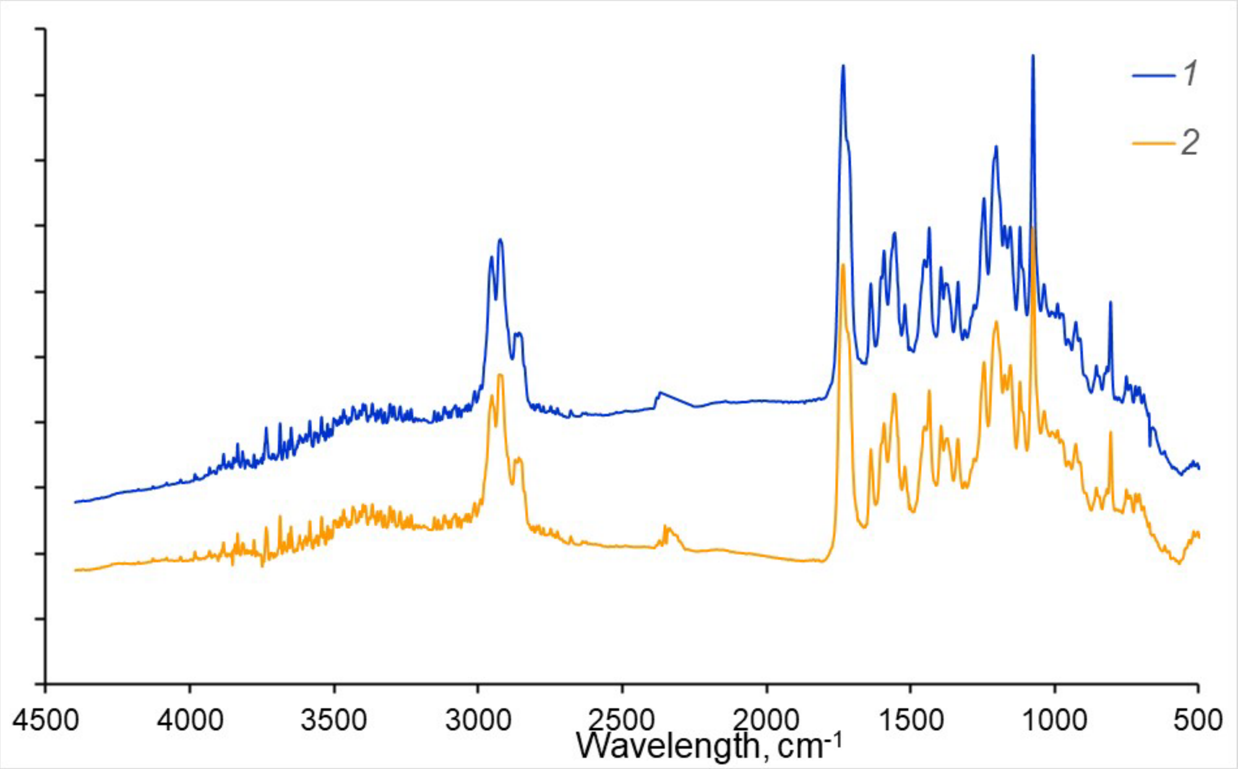

Figure S2. IR spectra of Cu-C-e6 (*1*) for the initial state (blue line) and (*2*) after the RTE deposition (brown line).

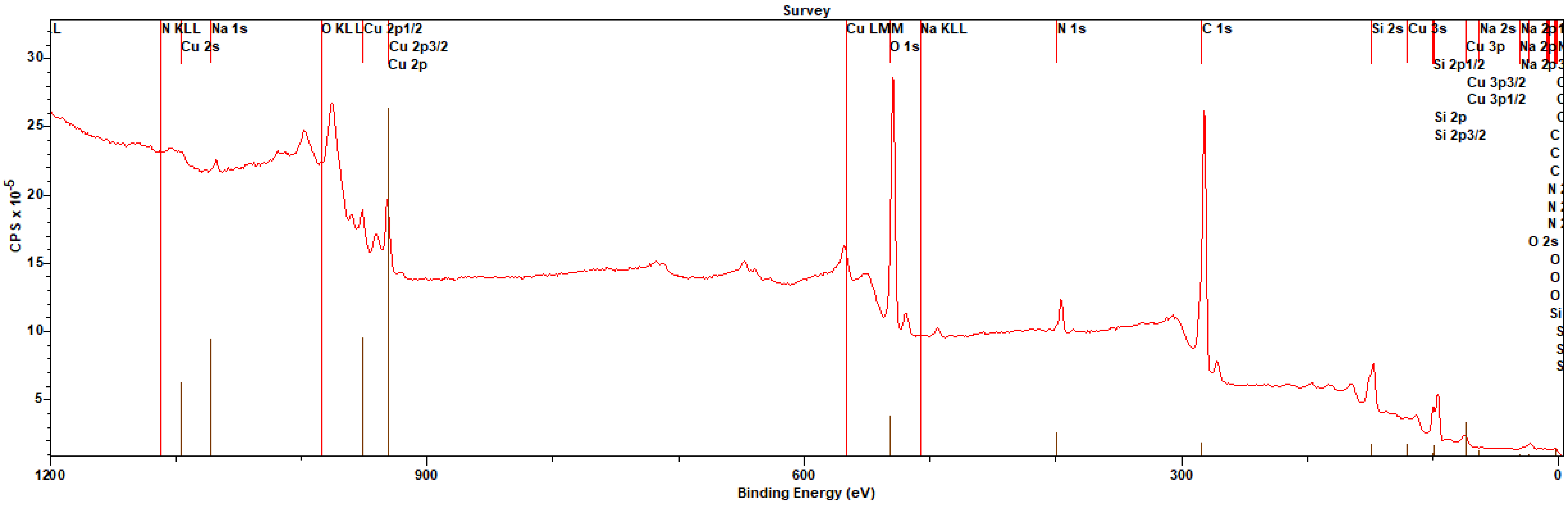

Figure S3. XPS spectrum of the initial chlorin Cu-C-e6.

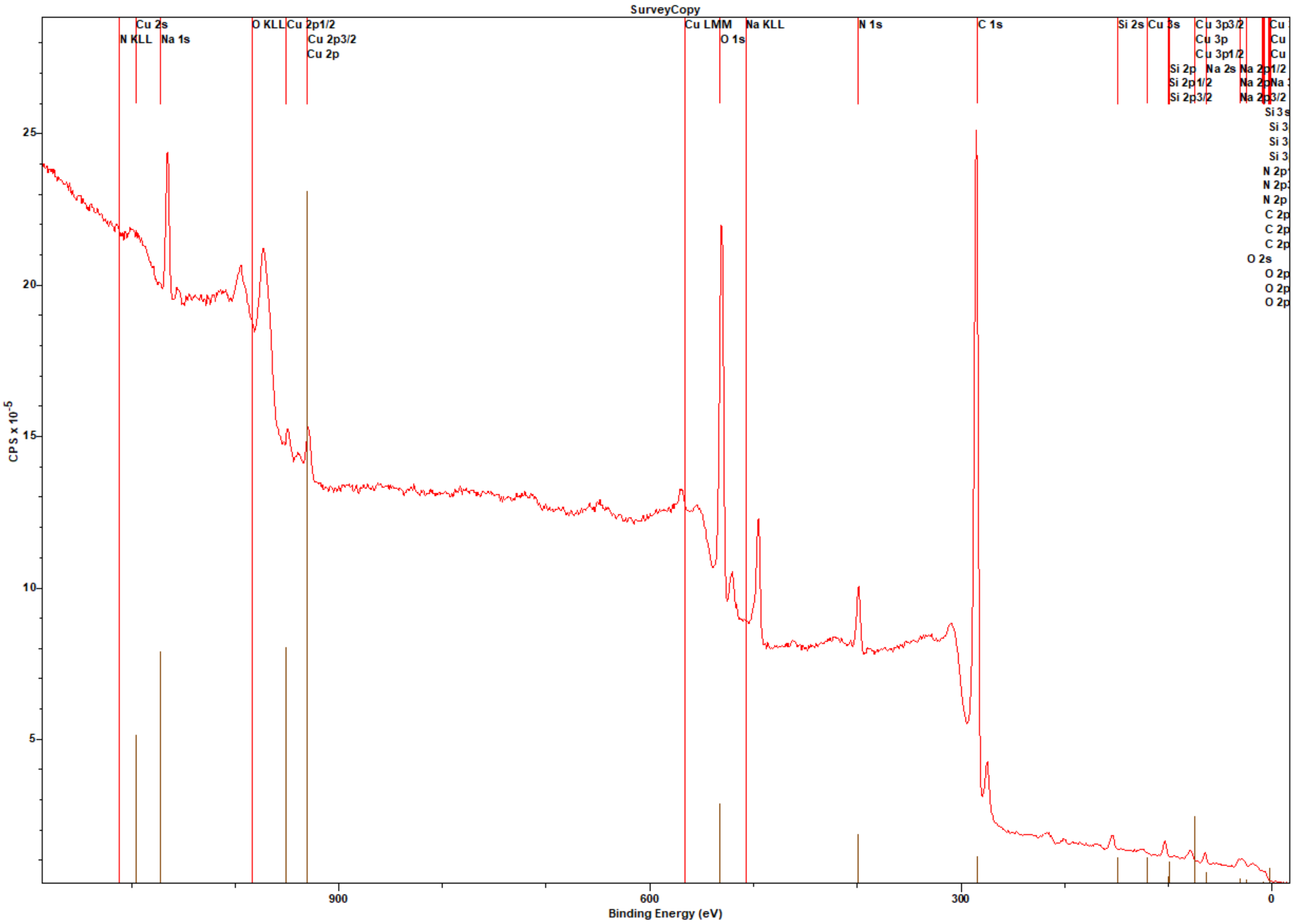

Figure S4. XPS spectrum of the chlorin Cu-C-e6 after the RTE deposition

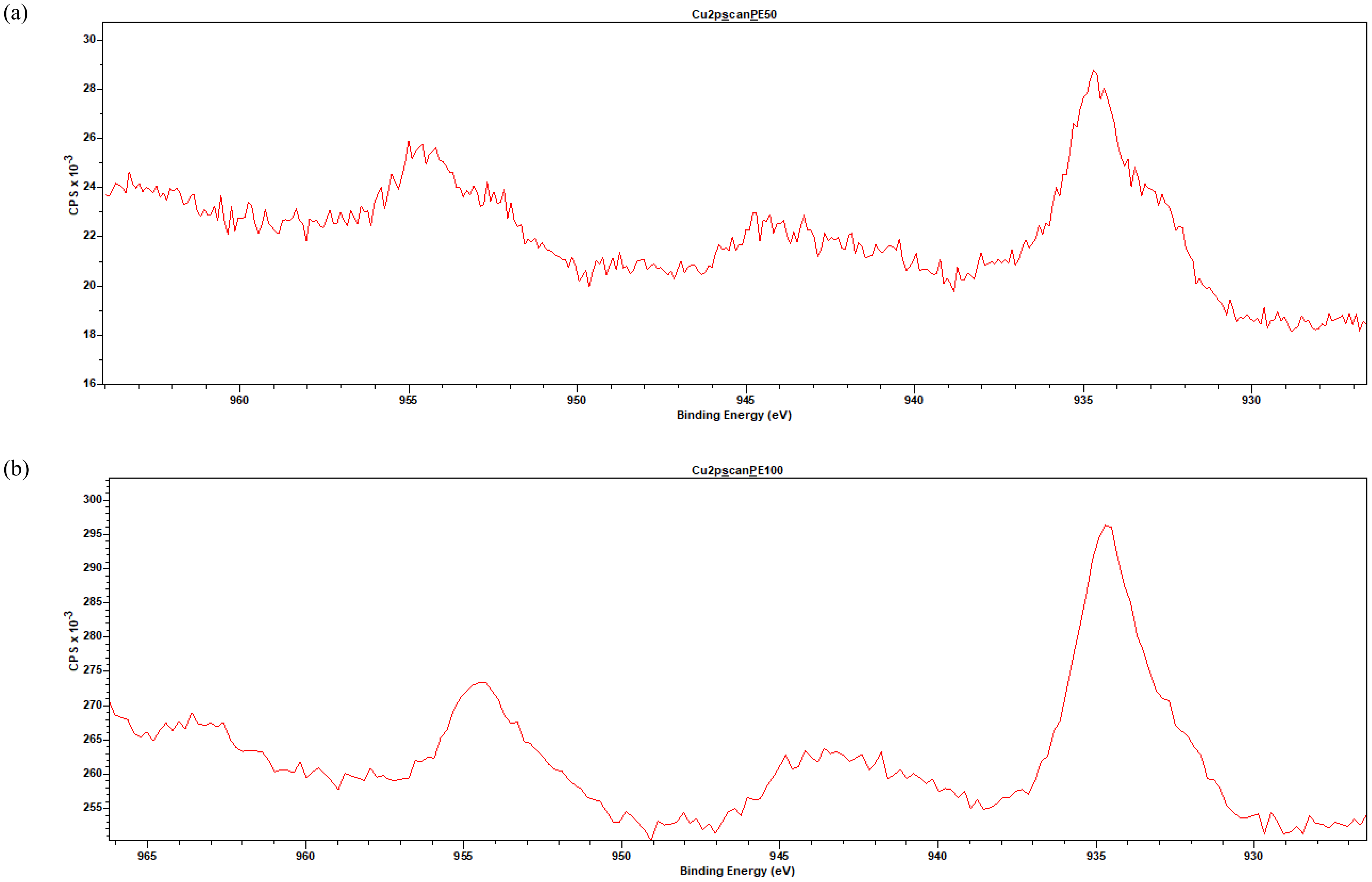

Figure S5. XPS spectra of the chlorin Cu2p band for (a) the initial and (b) after the RTE deposition of the dye

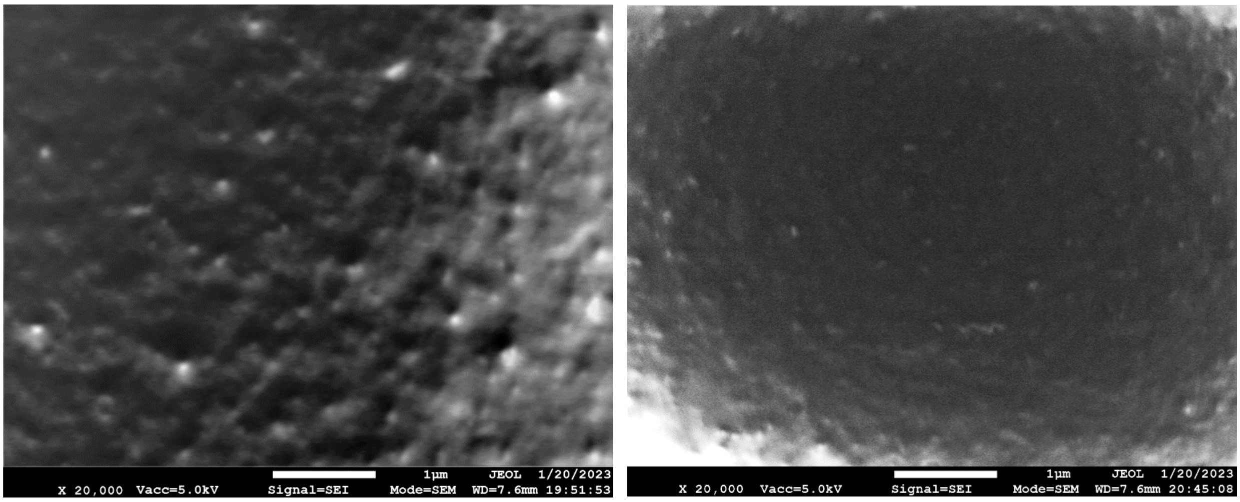

(**a**) (**b**)

Figure S6. SEM microphotographs of the RTE deposited layers of (a) Cu-PP-IX and (b) Cu-C-e6.

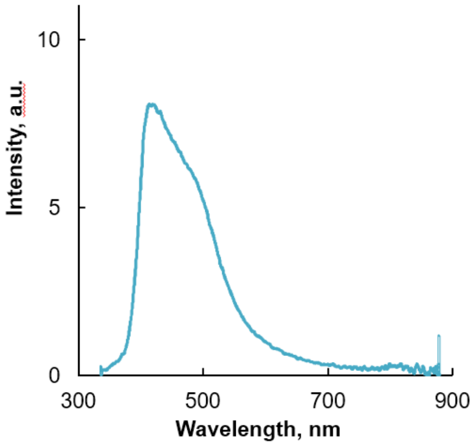

Figure S7. EL spectrum of the ITO/PEDOT: PSS/PVK: PBD/TPBi/LiF/Al device without Cu (II) porphyrinate recorded at 15 V.
